# Cumulative Live Birth Rates According to Maternal Body Mass Index After First Ovarian Stimulation for *in vitro* Fertilization: A Single Center Analysis of 14,782 Patients

**DOI:** 10.3389/fendo.2020.00149

**Published:** 2020-04-09

**Authors:** Xia Xue, Wenhao Shi, Hanying Zhou, Li Tian, Zhenghao Zhao, Dangxia Zhou, Juanzi Shi

**Affiliations:** ^1^Department of Pathology, Medical School, Xi'an Jiaotong University, Xi'an, China; ^2^The Assisted Reproduction Center, Northwest Women's and Children's Hospital, Affiliated With Xi'an Jiaotong University, Xi'an, China

**Keywords:** BMI, cumulative live birth rate, *in vitro* fertilization, overweight, underweight

## Abstract

**Objective:** To investigate the cumulative live birth rates (CLBR) according to body mass index (BMI) in women undergoing their first *in vitro* fertilization (IVF).

**Design:** Retrospective cohort analysis.

**Setting:** An IVF clinic in a public hospital.

**Patients:** This is a retrospective study of 14,782 patients undergoing their first fresh IVF cycles and subsequent frozen embryo transfers in our clinic from January 2014 to January 2017. The follow-up for CLBR continued until January 2019. Patients with a BMI <18.5 kg/m^2^ were considered to be underweight and those with a BMI > 24 kg/m^2^ were considered to be overweight. Patients with a BMI ≥ 28 kg/m^2^ were considered to be obese.

**Intervention(s):** None.

**Primary Outcome Measure:** The primary outcome was cumulative live birth rate (CLBR).

**Result(s):** This study illustrated the “inverted U shape” associations between body weight and IVF outcome (CLBR). The turning points in threshold analysis, as found by an automatic search, were BMIs of 18.5 and 30.4 kg/m^2^. The main finding of this retrospective data analysis is that the CLBR increased in underweight women, plateaued for normal weight and overweight women with a BMI between 18.5 and 30.4 kg/m^2^, and decreased in obese women.

**Conclusion(s):** The data suggested an “inverted U shape” association between BMI and CLBR. The CLBR increases in underweight women, plateaus in normal weight and overweight women, and then decreases in obese women.

## Introduction

Numerous studies have shown that a body mass index (BMI) that is either too high or too low is associated with a reduced probability of achieving pregnancy in women undergoing assisted reproductive technology (ART) (1). However, most of these studies are limited by being cycle-based (2–5), in which they only studied fresh cycles and reported the outcome in terms of live births per fresh cycle or embryo transfer. There cannot be a complete measure of an *in vitro* fertilization (IVF) treatment's success without a comprehensive analysis of the frozen embryo transfer (FET). In addition to affecting other aspects of the body's functions (6), BMI has an impact on the female reproductive system, both pre- and post-pregnancy. In this respect, a patient-based cumulative live birth rate (CLBR) that includes all fresh and frozen embryo transfers is a more suitable and more comprehensive measurement when reporting outcomes of ART (7). Before now, there were few reports using CLBR as the primary outcome measurement for the impact of BMI on IVF.

Current studies have adopted WHO classification criteria for BMI classification. The cutoffs for underweight (<18.5 kg/m^2^), overweight (BMI 25–30 kg/m^2^), and obese (BMI >30 kg/m^2^) were adopted to define a different population (8). However, we are not sure whether the threshold of BMI impact on CLBR is consistent with this criterion. Continuous variable data of BMI impact on CLBR is still not illustrated. There is still a lack of effective information, especially regarding patients who fall outside the normal BMI range in weight counseling before IVF.

Taking into account all the above-mentioned details, we set out to examine the relationship between BMI and first-time CLBR after a single stimulation among women undergoing IVF. Furthermore, we would like to figure out the turning point of the BMI's impact on CLBR, which can provide a valuable counseling resource for infertile women with abnormal BMIs.

## Materials and Methods

This is a retrospective study of 14,782 patients undergoing first fresh IVF/intracytoplasmic sperm injection (ICSI) cycles with subsequent frozen embryo transfer (FET) in our clinic from January 2014 to January 2017. The follow-up for CLBR continued for two years until January 2019. The study was approved by the Ethics Committee for the Clinical Application of Human Assisted Reproductive Technology of Northwest Women's and Children's Hospital (No. 2018002).

### Patients' Inclusion Criteria

All first fresh IVF/ICSI patients (*n* = 14,782) who underwent subsequent FET from the same fresh oocyte retrieval in our clinic between January 2014 and January 2017 were included in the analysis.

The following cycles were excluded: (1) donated oocyte cycles, *n* = 14; (2) pre-implantation genetic testing (PGT) cycles, *n* = 96; (3) cycles that did not achieve live birth, with frozen embryos still in retrieval during this period, *n* = 292; (4) cycles lost to follow-up, *n* = 30; (5) cycles with induced abortion, *n* = 15; (6) invalid BMI value, *n* = 132.

**Figure 1:**
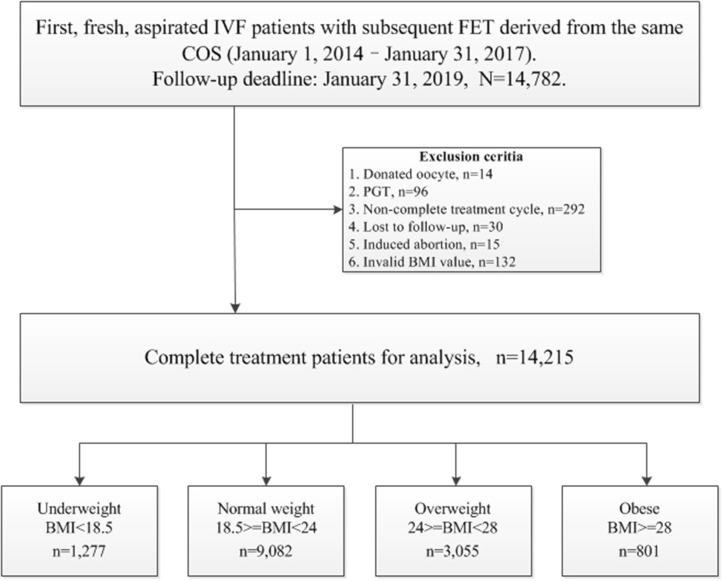
Flow chart and data processing (BMI, kg/m^2^).

The patients' BMIs were calculated as reported weight in kilograms per meter squared (reported height) at time of IVF start (before stimulation). The criteria for BMI categories were consistent with the international classifications of the World Health Organization (WHO), and adjusted according to the characteristics of an Asian population, which in general has a lower BMI than observed in non-Asian populations, with the BMI distribution shifted to the left (8). Thus, the patients with a BMI <18.5 kg/m^2^ were considered to be underweight, those with a BMI > 24 kg/m^2^ were considered to be overweight, and those with a BMI ≥ 28 kg/m^2^ were considered to be obese. Finally, 14,215 patients were studied for subsequent analysis. Flowchart and data processing are displayed in Figure 1. Demographics and basal characteristics of patients are presented in Table 1.

**Table 1:** Demographics and basal characteristics.

**Female BMI group (kg/m^**2**^)**	** <18.5**	****> = **18.5, <24**	****> = **24, <28**	****> = **28**	***P*-value**
*N*	1,277	9,082	3,055	801	
Age of female	28.97 ± 4.15	30.24 ± 4.65	30.90 ± 5.08	30.47 ± 4.79	<0.001
Age of female					<0.001
< =30	898 (70.32%)	5,407 (59.54%)	1,637 (53.58%)	468 (58.43%)	
>30, < =35	281 (22.00%)	2,467 (27.16%)	894 (29.26%)	212 (26.47%)	
>35, < =40	76 (5.95%)	887 (9.77%)	347 (11.36%)	90 (11.24%)	
>40	22 (1.72%)	321 (3.53%)	177 (5.79%)	31 (3.87%)	
Basal FSH (IU/ml)	7.46 ± 3.46	7.08 ± 3.04	6.75 ± 3.29	6.35 ± 1.92	<0.001
AFC	12.37 ± 5.45	12.32 ± 5.83	13.29 ± 6.68	14.44 ± 7.14	<0.001
Type of infertility					<0.001
Primary	855 (66.95%)	5,229 (57.58%)	1,638 (53.62%)	455 (56.80%)	
Secondary	422 (33.05%)	3,853 (42.42%)	1,417 (46.38%)	346 (43.20%)	
Length of infertility, year					<0.001
< =2	529 (41.88%)	3,657 (40.68%)	1,083 (35.83%)	224 (28.18%)	
>2, < =5	565 (44.73%)	3,800 (42.27%)	1,295 (42.84%)	368 (46.29%)	
>5	169 (13.38%)	1,532 (17.04%)	645 (21.34%)	203 (25.53%)	
Year of treatment					<0.001
2014	398 (31.17%)	2,594 (28.56%)	833 (27.27%)	192 (23.97%)	
2015	405 (31.71%)	2,848 (31.36%)	911 (29.82%)	258 (32.21%)	
2016–2017.01	474 (37.12%)	3,640 (40.08%)	1,311 (42.91%)	351 (43.82%)	
Gravidity					<0.001
0	849 (66.48%)	5,190 (57.16%)	1,622 (53.11%)	453 (56.55%)	
1	248 (19.42%)	1,976 (21.76%)	678 (22.20%)	186 (23.22%)	
>=2	180 (14.10%)	1,914 (21.08%)	754 (24.69%)	162 (20.22%)	
Parity					<0.001
0	1,194 (93.50%)	7,907 (87.06%)	2,529 (82.81%)	686 (85.64%)	
1	80 (6.26%)	1,072 (11.80%)	457 (14.96%)	97 (12.11%)	
>=2	3 (0.23%)	103 (1.13%)	68 (2.23%)	18 (2.25%)	
Main etiology					<0.001
Tubal factor	788 (62.10%)	5,881 (65.24%)	1,926 (63.67%)	482 (60.71%)	
Ovarian factor	79 (6.23%)	697 (7.73%)	438 (14.48%)	139 (17.51%)	
Male factor	190 (14.97%)	1,155 (12.81%)	287 (9.49%)	72 (9.07%)	
Endometriosis	33 (2.60%)	154 (1.71%)	43 (1.42%)	8 (1.01%)	
Uterine factor	21 (1.65%)	181 (2.01%)	56 (1.85%)	11 (1.39%)	
Other reasons	158 (12.45%)	946 (10.49%)	275 (9.09%)	82 (10.33%)	
OS protocol					0.001
GnRH agonist	1,122 (88.21%)	7,877 (86.91%)	2,579 (84.61%)	664 (83.10%)	
GnRH antagonist	123 (9.67%)	953 (10.52%)	382 (12.53%)	110 (13.77%)	
Other	27 (2.12%)	233 (2.57%)	87 (2.85%)	25 (3.13%)	
FSH start dose IU					<0.001
< =150	200 (25.67%)	1,094 (19.04%)	304 (15.72%)	50 (10.31%)	
>150, < =300	551 (70.73%)	4,398 (76.55%)	1,522 (78.70%)	399 (82.27%)	
>300	28 (3.59%)	253 (4.40%)	108 (5.58%)	36 (7.42%)	
Total Gn dose IU	2,247.88 ± 927.12	2,320.32 ± 976.42	2,546.62 ± 1,034.56	2,784.52 ± 1,054.73	<0.001
Gn type					0.383
Recombinant-FSH	724 (56.83%)	5,298 (58.77%)	1,742 (57.36%)	466 (58.47%)	
Urinary –FSH	550 (43.17%)	3,717 (41.23%)	1,295 (42.64%)	331 (41.53%)	
Female smoking					0.036
Yes	0 (0.00%)	7 (0.08%)	5 (0.16%)	3 (0.37%)	
No	1,277 (100.00%)	9,075 (99.92%)	3,050 (99.84%)	798 (99.63%)	

Mean + SD/N (%).

AFC, antral follicle count; BMI, body mass index; FSH, follicle stimulating hormone; Gn, Gonadotropin; GnRH, Gonadotropin-releasing hormone; OS, ovarian stimulation.

*Created by EmpowerStats (www.empowerstats.com) and R*.

### IVF Treatment

The protocol for ovarian stimulation (OS) was determined individually according to the patient's age, BMI, basal follicle-stimulating hormone, and antral follicle count (AFC). Most patients were treated with recombinant and/or urinary gonadotrophins (Gonal-F/PUREGON/Urofollitropin) in a long Gonadotropin-releasing hormone (GnRH) agonist or a GnRH antagonist protocol followed by IVF or ICSI. For women with diminished ovarian reserves, the mild ovulation protocol or luteal phase ovarian stimulation was attempted. Human menopausal gonadotrophin (HMG, Li Zhu, China) was added according to the patients' response to stimulation. Four thousands to ten thousands units of human chorionic gonadotrophin (hCG), or 250 μg r-hCG (Merck Seronoy S.p.A.), were administered when two to three follicles were >17 mm. Oocyte retrieval was performed 36 h later by transvaginal ultrasonography-guided aspiration. The ovarian stimulation parameters for each group are displayed in Table 1.

The procedures for oocyte extraction, embryo culture and the embryo scoring system were described in our previous studies (9, 10). Grades 1–3 were considered useable embryos on day 3, and good-quality embryos were Grades 1–2. All IVF/intracytoplasmic sperm injection (ICSI) embryo transfer (ET) procedures were performed according to the following principles: embryo transfers were carried out on days 3 or 5 in fresh cycles; in cases with sufficient high-quality embryos (more than 3–4) on day 3, blastocyst transfer on day 5 was practiced; after fresh embryo transfer, patients' surplus embryos were vitrified; in cases of unsuccessful implantation during the fresh cycle, FET was carried out using surplus embryos; for luteal phase support, progesterone injection (60 mg/day) was started after oocyte retrieval and maintained until a negative serum beta-hCG or the eighth week of pregnancy.

### Primary Outcome Measurement

The primary outcome was CLBR, which was defined as at least one live birth resulting from one aspirated ART cycle in the fresh transfer or subsequent FET in relation to the number of oocytes retrieved. CLBR was evaluated by adding the pregnancies and live births achieved in the FETs to the ones obtained in the fresh cycle. Only the first delivery was considered in the analysis. One treatment cycle is defined as an oocyte retrieval and all transfers, fresh and frozen/thawed, derived from that ovarian stimulation. One complete treatment cycle refers to a treatment cycle that achieved live birth or a treatment cycle that transferred all embryos but failed to achieve live birth.

### Statistical Analysis

The data processing and statistical analysis were conducted by EmpowerStats software (www.empowerstats.com, version R.3.4.3) and statistical software packages R. Multivariate. Logistic regression analyses were performed to evaluate the association between BMI and CLBR, with adjustments made for important co-variables and potential confounding factors (treatment year, patient's age, AFC, basal FSH, smoking, type of infertility, length of infertility, gravidity, parity, main etiology, stimulation protocol, total Gn dose, and starting FSH dose). A generalized additive model, where the outcome was the CLBR and the explanatory variable was a continuous BMI, was used to check for trend. A threshold analysis of BMI associated with CLBR was also performed.

## Results

Demographics and basal characteristics of IVF patients vary in Table 1. Obviously, there were significant differences among infertility-related parameters and anthropometrical variables by dividing patients according to BMI. Young women (<35 years) were more likely to be underweight, have < =2 years of infertility, have tubal factor infertility, use the GnRH antagonist protocol, receive a lower dose of Gn, and so on. These imbalanced variables may act as confounding factors and were adjusted in subsequent analysis. Statistically speaking, the CLBR significantly decreased among underweight (66.41%), normal weight (65.79%), overweight (61.08%), and obese (56.30%), as well as the number of day 3 embryos and the number of oocytes retrievals (Table 2). The oocyte output rate (oocytes retrieved/antral follicle count) also statistically significantly decreased among underweight (96.40%), normal weight (95.63%), overweight (85.89%), and obese (72.99%).

**Table 2:** Oocytes and embryo parameters and cumulative live birth rates.

**Female BMI group (kg/m^**2**^)**	** <18.5**	****> = **18.5, <24**	****> = **24, <28**	****> = **28**	***P*-value**
*N*	1,277	9,082	3,055	801	
No. of oocytes	11.99 ± 6.96	11.80 ± 6.91	11.45 ± 7.05	10.93 ± 6.75	<0.001
No. of cleavage	9.31 ± 5.79	9.16 ± 5.84	8.80 ± 5.89	8.52 ± 5.87	<0.001
No. 2PN	7.40 ± 4.76	7.21 ± 4.69	6.87 ± 4.69	6.55 ± 4.48	<0.001
No. of day 3 useable embryos	6.16 ± 4.27	6.01 ± 4.23	5.70 ± 4.29	5.42 ± 4.15	<0.001
No. of day 3 good quality embryos	3.67 ± 3.33	3.66 ± 3.31	3.50 ± 3.35	3.25 ± 3.18	0.001
No. of oocytes/AFC	96.40%	95.63%	85.89%	72.99%	<0.001
Cumulative live birth (rate %)	848 (66.41%)	5,975 (65.79%)	1,866 (61.08%)	451 (56.30%)	<0.001

AFC, antral follicle count; PN, pronucleus.

*Mean + SD/N (%), Created by EmpowerStats (www.empowerstats.com) and R*.

A multiple variables regression analysis was performed, taking into account the fact that variables may act as confounding factors to the data described in Table 1. The unadjusted and adjusted odds ratios (OR) of CLBR with a 95% confidence interval (CI) in different models are shown in Table 3. It was found that the BMI of underweight patients had no statistically significant effect on the CLBR (OR 0.92, 95% CI 0.80–1.04, *p* = 0.1902) compared with the normal weight group when adjusted for female age, AFC, main etiology, and OS protocol. The CLBR in overweight patients decreased significantly (OR 0.82, 95% CI 0.74–0.89, *p* <0.0001) compared with normal weight patients when adjusted for female age, AFC, main etiology, and OS protocol. However, the CLBR in the obese patients decreased by about 40% (OR 0.60, 95% CI 0.51–0.70, *p* <0.0001) when compared with the normal weight group in model 1.

**Table 3:** Logistic regression analysis for cumulative live birth rates in BMI groups.

	**Non-adjusted OR (95% CI) *P*-value**	**Adjust I** **OR (95% CI) *P*-value**	**Adjust II OR** **(95%CI) *P*-value**
Female BMI group (kg/m^2^)			
>=18.5, <24	1 (ref)	1 (ref)	1 (ref)
<18.5	1.03 (0.91, 1.16) 0.6637	0.92 (0.80, 1.04) 0.1902	0.86 (0.73, 1.02) 0.0888
>=24, <28	0.82 (0.75, 0.89) <0.0001	0.82 (0.74, 0.89) <0.0001	0.86 (0.76, 0.97) 0.0118
>=28	0.67 (0.58, 0.78) <0.0001	0.60 (0.51, 0.70) <0.0001	0.63 (0.51, 0.77) <0.0001

OR (95% CI) P-value.

Adjusted I for female age, AFC, main etiology, OS protocol.

Adjusted II for year of treatment, patient's age, AFC, basal FSH, smoking, type of infertility, length of infertility, gravidity, parity, main etiology, OS protocol, Gn total dose, and FSH start dose.

*Created by EmpowerStats (www.empowerstats.com) and R*.

**Figure 2:**
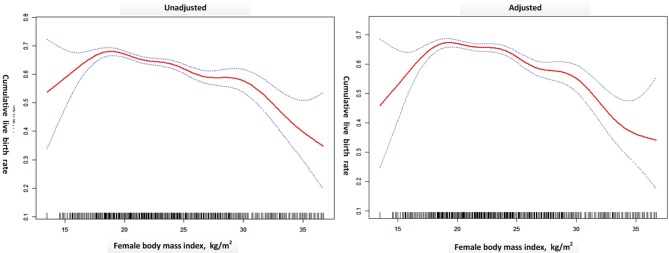
A generalized additive model was built where the outcome was the CLBR and the explanatory variable was BMI (red dots). The blue dotted lines are the 95% confidence intervals. The right model was adjusted for treatment year, patient's age, AFC, basal FSH, smoking habits, type of infertility, length of infertility, gravidity, parity, main etiology, OS protocol, Gn total dose, Gn type, and FSH start dose. Created by EmpowerStats (www.empowerstats.com) and R.

A generalized additive model (Figure 2) was built where the outcome was the CLBR and the explanatory variable was BMI (red dots). The blue dotted lines are the 95% confidence intervals. The right model was adjusted for treatment year, patient's age, AFC, basal FSH, smoking habits, type of infertility, length of infertility, gravidity, parity, main etiology, OS protocol, Gn total dose, Gn type, and starting FSH dose. The CLBR increased linearly from a BMI of 15 kg/m^2^ to a BMI of ~18 kg/m^2^, then showed a slowly declining segment. When BMI exceeded ~30 kg/m^2^, the CLBR fell sharply until the nadir. The association between CLBR and BMI looks like an inverted U-shape. The turning points (BMI 18.5 and 30.4 kg/m^2^) were determined using threshold analysis (Table 4).

**Table 4:** Threshold analysis of BMI associated with cumulative live birth rate.

**Outcome**	**CLBR**
Model I	
One-line slope	0.96 (0.95, 0.97) <0.0001
Model II	
Turing points (K1,K2)	18.5, 30.4
< K1 Slope 1	1.26 (1.12, 1.41) <0.0001
K1-K2 Slope 2	0.96 (0.94, 0.97) <0.0001
>K2 Slope 3	0.88 (0.74, 1.05) 0.1589
Slope 1-Slope 2	1.31 (1.16, 1.48) <0.0001
Slope 3-Slope 2	0.92 (0.78, 1.10) 0.3648
LRT test	<0.001

Results in table: OR (95% CI) P-value. Adjusted for female age, AFC, main etiology, OS protocol.

*Created by EmpowerStats (www.empowerstats.com) and R*.

**Figure 3:**
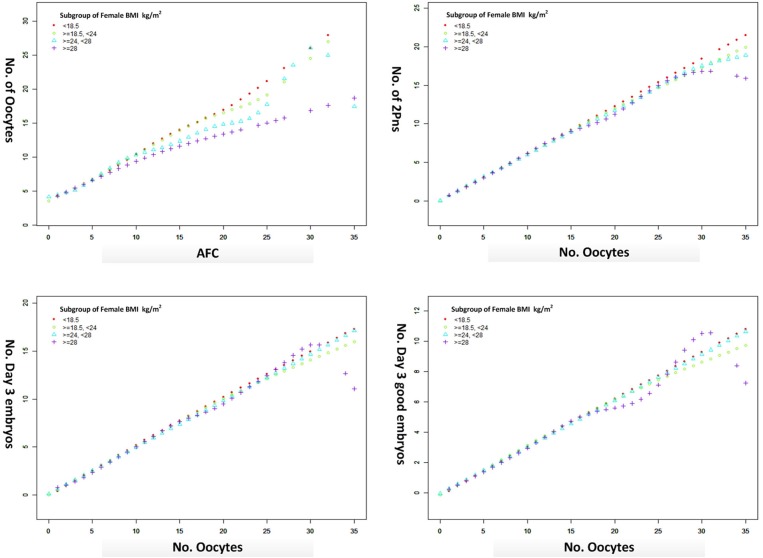
The oocyte retrieval (number of oocytes/AFC) and embryo development (number of 2pn, day 3 embryos and good embryos/no. oocytes) in different subgroups of BMI from a generalized additive model, adjusted for female age, main etiology, and OS protocol.

The oocyte retrieval (number of oocytes/AFC) and embryo development (number of 2pn, day 3 embryos and good embryos/number of oocytes) in different subgroups of BMI were displayed in Figure 3. The differences between BMI subgroups were mainly expressed in oocyte retrieval, rather than embryo development indicators, such as 2pns, day 3 embryos, and good day 3 embryos per oocyte. The subgroup analysis was performed according to the ranges of AFC, patient's age, and stimulation protocol in Table 5, in which the CLBR showed a trend similar to the general trend in all subgroups with the addition of women over 35. The multivariable fractional polynomials analysis was conducted in Table S1, which could be helpful to highlight which parameters better predict the CLBR.

**Table 5:** Subgroup analysis of CLBR according to AFC, maternal age, and stimulation protocol.

**BMI (kg/m^**2**^)**	***N***	****> = **18.5, <24**	** <18.5**	****> = **24, <28**	****> = **28**
		**Ref**.	**OR (95% CI) *P-*value**	**OR (95% CI) *P-*value**	**OR (95% CI) *P*-value**
**Age of female**					
< =30	8,410	1	0.94 (0.80, 1.09) 0.4031	0.81 (0.72, 0.92) 0.0008	0.57 (0.47, 0.69) <0.0001
>30, < =35	3,854	1	0.81 (0.63, 1.04) 0.0974	0.96 (0.82, 1.13) 0.6590	0.73 (0.55, 0.96) 0.0271
>35, < =40	1,400	1	0.94 (0.58, 1.51) 0.8010	0.89 (0.69, 1.15) 0.3702	1.04 (0.67, 1.62) 0.8468
>40	551	1	1.21 (0.34, 4.29) 0.7658	1.32 (0.77, 2.27) 0.3106	1.48 (0.53, 4.08) 0.4528
**AFC**					
< =4	1,220	1	1.26 (0.76, 2.08) 0.3654	0.81 (0.59, 1.12) 0.2013	0.61 (0.34, 1.12) 0.1124
>4, < =9	3,542	1	0.93 (0.74, 1.17) 0.5372	0.90 (0.76, 1.06) 0.2055	0.86 (0.62, 1.20) 0.3684
>9, < =15	4,954	1	0.96 (0.78, 1.18) 0.6868	0.75 (0.65, 0.88) 0.0004	0.71 (0.53, 0.94) 0.0167
>15	4,499	1	1.07 (0.82, 1.41) 0.6040	0.69 (0.59, 0.81) <0.0001	0.43 (0.34, 0.54) <0.0001
**OS protocol**					
GnRH agonist	12,242	1	0.99 (0.86, 1.13) 0.8828	0.84 (0.76, 0.92) 0.0003	0.68 (0.58, 0.80) <0.0001
GnRH antagonist	1,568	1	1.01 (0.69, 1.47) 0.9758	0.80 (0.63, 1.02) 0.0743	0.67 (0.45, 1.01) 0.0535
Other	372	1	2.65 (1.11, 6.35) 0.0289	0.61 (0.28, 1.33) 0.2127	1.32 (0.47, 3.75) 0.5969
**Fertilization approach**					
IVF	10,698	1	1.08 (0.93, 1.25) 0.3150	0.85 (0.77, 0.94) 0.0014	0.72 (0.61, 0.86) 0.0001
ICSI	3,517	1	0.90 (0.71, 1.14) 0.3930	0.71 (0.59, 0.84) <0.0001	0.52 (0.38, 0.70) <0.0001

## Discussion

The main finding of this retrospective data analysis is that the CLBR increased among underweight women, plateaued in normal weight and overweight women, and then decreased in obese women. The highest CLBR was observed among women with a BMI of 18.5 kg/m^2^, surrounded by normal weight and underweight women. Another finding was that oocyte output rate decreased as BMI increased. However, there is no difference in the embryo development indicators.

In 2004, Fedorcsák et al. (11) first reported the CLBR of underweight and overweight women in a large cohort of women who underwent IVF, which is consistent with the present study. Rather than calculating CLBR with the Kaplan–Meier method after three cycles of IVF like Fedorcsak, our calculation was a patient-based outcome measurement stressing the first fresh aspirated IVF treatment, including all fresh and frozen embryo transfers. Moreover, the spline smoothing plot of CLBR according to BMI was first presented to illustrate their relationship.

The “inverted U shape” associations between BMI and IVF outcome have been shown in several studies (2, 4, 12, 13). While obesity has been demonstrated to impair pregnancy outcome, more issues focus on underweight and overweight women (24–28 kg/m^2^). Our results suggested that being underweight had no significant effect on CLBR and being overweight had a slight impact and decreased CLBR, which is consistent with the results from the 2008–2013 Society for Assisted Reproductive Technology registry in the United States (2, 4). Unfortunately, in some studies (14–16), the obese group was included in the overweight group for analysis rather than the subdivision analysis. Though the mechanism of the negative effect of BMI on IVF outcome is unclear, the relationship may have a threshold effect on the CLBR by accumulative BMI. Our study figured out the cut-off value of 30.4 kg/m^2^, which means the IVF outcome of women with a BMI of more than 30.4 kg/m^2^ would be severely impaired by excessive BMI. At the other body weight extreme, women with a BMI of less than 18.5 kg/m^2^ would have impaired CLBR due to low BMI.

Most studies (4, 17–19) failed to demonstrate the association between low BMI and impaired IVF outcome. However, Cai et al. (3) recently concluded that low BMI is associated with negative outcomes in fresh transfer cycles, especially in women of advanced age. Obviously, in Cai's study only fresh transfer cycles were included, and frozen embryo transfers and cases with canceled fresh transfers were both excluded. Biases of population selection may cause inconsistency with our results. In addition, the outcome measurement in Cai's study adopted the simple live birth rate rather than the CLBR used in our study. Contrary to the results of Cai's study in the same Chinese population, low BMI was not associated with reduced CLBR (OR 0.89, 95% CI 0.75–1.06, *p* = 0.1891). Instead, the highest CLBR was observed among women with a BMI of 18.5 kg/m^2^.

Maternal BMI is significantly associated with CLBR in the patients with first IVF/ICSI treatment. The CLBR remains relatively constant in IVF patients in the normal and overweight range (BMI 18.5–30.4). Therefore, for these patients, it is not necessary to be concerned about the impact of BMI on CLBR. Being underweight may have a limited impact on IVF success (namely CLBR), however, and gaining weight still improves the chances of CLBR (about 26% per unit of BMI). Being obese has a significant impact on IVF success, with a CLBR loss of about 12% for every one unit increase of BMI. This provides specific and effective data in support of weight counseling for infertile women before IVF treatment.

Our data suggested that the oocyte output rate (number of oocytes/AFC) was damaged due to the excessive BMI. However, there was no difference in the embryo development indicators. Consistent with the literature, obesity was associated with ovulatory dysfunction and higher doses of ovarian stimulation in IVF (20, 21), leading to a lower chance of oocyte retrieval. Although the evidence from both clinical data and animal studies suggests obesity negatively impacts the developmental competence of oocytes (22, 23), there are still questions about the pathophysiology underlying these findings because not all studies took into account the damage in embryo quality (24, 25).

Limitations are related to the retrospective nature of the study, and analysis from a single center also weakens the universality of our observations. The results of the study should also be interpreted with caution when considering other populations and areas because Asian populations in general have a lower BMI than observed in non-Asian populations, with a BMI distribution shifted to the left (8). The variance of BMI during IVF treatment and pregnancy, which was not involved in the study, may also be a potential confounding impact on the live birth.

## Conclusion

In conclusion, there is an “inverted U shape” association between body weight and IVF outcome (CLBR). CLBR increased among underweight women, plateaued in normal weight and overweight women with a BMI of 18.5–30.4, and then decreased in obese women proportionally to the accumulation of BMI. The present study may therefore be helpful for IVF patients with abnormal BMI to predict pregnancy outcome (CLBR) when attending weight counseling before initiating IVF cycles.

## Data Availability Statement

All datasets generated for this study are included in the article/Supplementary Material.

## Author Contributions

XX: writing the article, study conception and design, and critical review of the article. WS, LT, ZZ, and HZ: data processing. DZ and JS: supervising the study. All authors approved the manuscript at submission format.

### Conflict of Interest

The authors declare that the research was conducted in the absence of any commercial or financial relationships that could be construed as a potential conflict of interest.

## References

[1] Sarais V Pagliardini L Rebonato G Papaleo E Candiani M Vigano P. A comprehensive analysis of body mass index effect on *in vitro* fertilization outcomes. Nutrients. (2016) 8:109. 10.3390/nu8030109 26907340 PMC4808839

[2] Provost MP Acharya KS Acharya CR Yeh JS Steward RG Eaton JL. Pregnancy outcomes decline with increasing body mass index: analysis of 239,127 fresh autologous *in vitro* fertilization cycles from the 2008-2010 Society for Assisted Reproductive Technology registry. Fertil Steril. (2016) 105:663–9. 10.1016/j.fertnstert.2015.11.008 26627120

[3] Cai J Liu L Zhang J Qiu H Jiang X Li P. Low body mass index compromises live birth rate in fresh transfer *in vitro* fertilization cycles: a retrospective study in a Chinese population. Fertil Steril. (2017) 107:422–9 e2. 10.1016/j.fertnstert.2016.10.029 27887711

[4] Kawwass JF Kulkarni AD Hipp HS Crawford S Kissin DM Jamieson DJ. Extremities of body mass index and their association with pregnancy outcomes in women undergoing *in vitro* fertilization in the United States. Fertil Steril. (2016) 106:1742–50. 10.1016/j.fertnstert.2016.08.028 27666564 PMC11056966

[5] Friedler S Cohen O Liberty G Saar-Ryss B Meltzer S Lazer T. Should high BMI be a reason for IVF treatment denial? Gynecol Endocrinol. (2017) 33:853–6. 10.1080/09513590.2017.1327042 28531369

[6] Imterat M Agarwal A Esteves SC Meyer J Harlev A. Impact of Body Mass Index on female fertility and ART outcomes. Panminerva Med. (2019) 61:58–67. 10.23736/S0031-0808.18.03490-0 29962181

[7] Maheshwari A McLernon D Bhattacharya S. Cumulative live birth rate: time for a consensus? Hum Reprod. (2015) 30:2703–7. 10.1093/humrep/dev263 26466912

[8] WHO Expert Consultation Appropriate body-mass index for Asian populations and its implications for policy and intervention strategies. Lancet. (2004) 363:157–63. 10.1016/S0140-6736(03)15268-3 14726171

[9] Shi W Zhang S Zhao W Xia X Wang M Wang H. Factors related to clinical pregnancy after vitrified-warmed embryo transfer: a retrospective and multivariate logistic regression analysis of 2313 transfer cycles. Hum Reprod. (2013) 28:1768–75. 10.1093/humrep/det094 23599130

[10] Shi W Xue X Zhang S Zhao W Liu S Zhou H. Perinatal and neonatal outcomes of 494 babies delivered from 972 vitrified embryo transfers. Fertil Steril. (2012) 97:1338–42. 10.1016/j.fertnstert.2012.02.051 22464083

[11] Fedorcsak P Dale PO Storeng R Ertzeid G Bjercke S Oldereid N. Impact of overweight and underweight on assisted reproduction treatment. Hum Reprod. (2004) 19:2523–8. 10.1093/humrep/deh485 15319380

[12] Kasim K Roshdy A. Body mass index and pregnancy outcome after assisted reproduction treatment. Int J Reprod Med. (2014) 2014:257974. 10.1155/2014/257974 25763394 PMC4334051

[13] Veleva Z Tiitinen A Vilska S Hyden-Granskog C Tomas C Martikainen H. High and low BMI increase the risk of miscarriage after IVF/ICSI and FET. Hum Reprod. (2008) 23:878–84. 10.1093/humrep/den017 18281684

[14] Supramaniam PR Mittal M McVeigh E Lim LN. The correlation between raised body mass index and assisted reproductive treatment outcomes: a systematic review and meta-analysis of the evidence. Reprod Health. (2018) 15:34. 10.1186/s12978-018-0481-z 29486787 PMC5830337

[15] Caillon H Freour T Bach-Ngohou K Colombel A Denis MG Barriere P. Effects of female increased body mass index on *in vitro* fertilization cycles outcome. Obes Res Clin Pract. (2015) 9:382–8. 10.1016/j.orcp.2015.02.009 25769458

[16] Purewal S Chapman SCE van den Akker OBA. A systematic review and meta-analysis of lifestyle and body mass index predictors of successful assisted reproductive technologies. J Psychosom Obstet Gynaecol. (2019) 40:2–18. 10.1080/0167482X.2017.1403418 29172958

[17] Lashen H Ledger W Bernal AL Barlow D Extremes of body mass do not adversely affect the outcome of superovulation and *in-vitro* fertilization. Hum Reprod. (1999) 14:712–5. 10.1093/humrep/14.3.712 10221701

[18] Wittemer C Ohl J Bailly M Bettahar-Lebugle K Nisand I. Does body mass index of infertile women have an impact on IVF procedure and outcome? J Assist Reprod Genet. (2000) 17:547–52. 10.1023/A:1009402130903 11209534 PMC3455453

[19] Singh N Gupta P Mittal S Malhotra N. Correlation of body mass index with outcome of *in vitro* fertilization in a developing country. Arch Gynecol Obstet. (2012) 285:259–63. 10.1007/s00404-011-2013-8 21792549

[20] Broughton DE Moley KH. Obesity and female infertility: potential mediators of obesity's impact. Fertil Steril. (2017) 107:840–7. 10.1016/j.fertnstert.2017.01.017 28292619

[21] Practice Committee of the American Society for Reproductive Medicine Obesity and reproduction: a committee opinion. Fertil Steril. (2015) 104:1116–26. 10.1016/j.fertnstert.2015.08.018 26434804

[22] Finger BJ Harvey AJ Green MP Gardner DK. Combined parental obesity negatively impacts preimplantation mouse embryo development, kinetics, morphology and metabolism. Hum Reprod. (2015) 30:2084–96. 10.1093/humrep/dev142 26089300

[23] Purcell SH Moley KH. The impact of obesity on egg quality. J Assist Reprod Genet. (2011) 28:517–24. 10.1007/s10815-011-9592-y 21625966 PMC3158259

[24] Metwally M Cutting R Tipton A Skull J Ledger WL Li TC. Effect of increased body mass index on oocyte and embryo quality in IVF patients. Reprod Biomed Online. (2007) 15:532–8. 10.1016/S1472-6483(10)60385-9 18044034

[25] Depalo R Garruti G Totaro I Panzarino M Vacca MP Giorgino F. Oocyte morphological abnormalities in overweight women undergoing *in vitro* fertilization cycles. Gynecol Endocrinol. (2011) 27:880–4. 10.3109/09513590.2011.569600 21500991

