# Supplementary material for: Cumulative Live Birth Rates According to Maternal Body Mass Index After First Ovarian Stimulation for in vitro Fertilization: A Single Center Analysis of 14,782 Patients
Source: Front Endocrinol (Lausanne). 2020 Apr 9;11:149. doi: 10.3389/fendo.2020.00149 (PMC7160227; doi:10.3389/fendo.2020.00149)
Supplement: Supplementary file 1 [file Table_1.pdf]

Supplementary Material

Table S1 The Multiple Fractional Polynomial model

	Estimate	Std error	OR	95%CI.low	95%CI.upp	P-value
(Intercept)	7.5747	2.2489	1948.349	23.7344	159939	0.0008
I((AFC/10)^0.5)	2.1633	0.1931	8.6999	5.958	12.7036	0
I((AFC/10)^3)	-0.0658	0.0117	0.9363	0.915	0.9581	0
I((Female age/10)^3)	0.1763	0.0324	1.1929	1.1194	1.2711	0
I((Female ag/10)^3 * log((X5/10)))	-0.1287	0.0213	0.8792	0.8433	0.9167	0
factor(OS protocol=GnRH antagonist)	0.0107	0.0977	1.0107	0.8346	1.2241	0.913
factor(OS protocol=other)	-0.64	0.2251	0.5273	0.3392	0.8197	0.0045
factor(Ovarian factor)	-0.2246	0.0911	0.7988	0.6682	0.955	0.0137
factor(Male factor)	0.1258	0.0789	1.134	0.9717	1.3236	0.1106
factor(Endometriosis)	-0.09	0.1939	0.914	0.625	1.3365	0.6427
factor(Uterine factor)	-0.2258	0.1767	0.7978	0.5643	1.128	0.2012
factor(Other reasons)	-0.0867	0.0812	0.917	0.7821	1.0752	0.2858
factor(FSH strart dose >150, <=300)	-0.16	0.0754	0.8521	0.7351	0.9878	0.0338
factor(FSH strart dose >300)	-0.4003	0.1562	0.6701	0.4934	0.9101	0.0104
log((Total Gn dose/1000))	2.0862	0.4913	8.0542	3.0751	21.0953	0
I((Total Gn dose/1000)^0.5)	-2.9728	0.6326	0.0512	0.0148	0.1768	0
I((BMI/10)^-2)	-8.1852	1.7844	0.0003	0	0.0092	0
I((BMI/10)^0.5)	-5.0122	0.9779	0.0067	0.001	0.0453	0
factor(No female smoking)	2.4616	1.1003	11.7233	1.3567	101.303	0.0253
factor(Year of treatment=2015)	-0.0069	0.0605	0.9932	0.8821	1.1182	0.9098
factor(Year of treatment=2016)	0.1132	0.0624	1.1199	0.991	1.2655	0.0694
factor(Year of treatment=2017)	-0.3266	0.2037	0.7214	0.4839	1.0754	0.1089
factor(Secondary infertility)	0.0711	0.0546	1.0737	0.9649	1.1949	0.1922
I((bFSH/10)^1)	-0.0844	0.0879	0.9191	0.7736	1.0919	0.3372
factor(Length of infertility <=2years)	-0.0485	0.0549	0.9527	0.8556	1.0608	0.377
factor(Length of infertility >2, <=5 years)	-0.1227	0.0727	0.8845	0.7671	1.0199	0.0914

AFC, antral follicle count; BMI: body mass index; bFSH, basic follicle stimulating hormone; FSH,

follicle stimulating hormone; Gn, Gonadotropin; GnRH, Gonadotropin releasing hormone; OS, ovarian stimulation.
